# Individual Differences in Personality Moderate the Effects of Perceived Group Deprivation on Violent Extremism: Evidence From a United Kingdom Nationally Representative Survey

**DOI:** 10.3389/fpsyg.2022.790770

**Published:** 2022-02-24

**Authors:** Bettina Rottweiler, Paul Gill

**Affiliations:** Security and Crime Science Department, University College London, London, United Kingdom

**Keywords:** group-based relative deprivation, violent extremism, trait entitlement, need for status, need for uniqueness, trait forgiveness, self-control, critical thinking

## Abstract

Numerous studies argue that perceived group deprivation is a risk factor for radicalization and violent extremism. Yet, the vast majority of individuals, who experience such circumstances do not become radicalized. By utilizing models with several interacting risk and protective factors, the present analysis specifies this relationship more concretely. In a large United Kingdom nationally representative survey (*n* = 1,500), we examine the effects of group-based relative deprivation on violent extremist attitudes and violent extremist intentions, and we test whether this relationship is contingent upon several individual differences in personality. The results show that stronger group-based injustices lead to increased support for and intentions to engage in violent extremism. However, some of the effects are much stronger for individuals who exhibit a stronger need for uniqueness and for status and who demonstrate higher levels of trait entitlement. Conversely, several effects are lessened for those individuals high in trait forgiveness, demonstrating a strong capacity for self-control and for those who are exerting critical as well as open-minded thinking styles, thus constituting buffering protective factors, which dampen the adverse effects of perceived group injustice on violent extremism. The results highlight the importance of considering (a) the interaction between individual dispositions and perceptions of contextual factors (b) the conditional and cumulative effects of various risk and protective factors and (c) the functional role of protective factors when risk factors are present. Collectively, these findings bring us one step closer to understanding who might be more vulnerable to violent extremism as well as how. Overall, the study suggests that preventing and countering violent extremism (P/CVE) programs must take account of the constellation of multiple factors that interact with (and sometimes enable or disable) one another and which can be targeted in preventions strategies.

## Introduction

Preventing the onset of violent radicalization is a key policy priority. Such interventions often require risk assessments to prioritize cases and allocate management plans tailored to the individuals’ needs. Risk assessment practice requires the best possible science yet the evidence behind several commonly used risk factors requires further work. This study focuses on one such factor: relative group deprivation. Several studies and conceptual models argue it is fundamental to how radicalization occurs (e.g., Borum, 2003; Moghaddam, 2005; McCauley and Moskalenko, 2008; Kruglanski et al., 2014) across different ideological contexts (Van den Bos, 2019; Kunst and Obaidi, 2020). However, the vast majority of individuals who experience such circumstances, do not become radicalized. Thus, other factors must concurrently be at play. The present study specifies these relationships more concretely and investigates whether the impact of relative deprivation on radicalization outcomes is contingent upon several individual differences in personality.

Relative group deprivation captures perceptions of injustice, discrimination and unfair treatment of one’s group. The in-group is considered to have less than what they are rightfully entitled to and to be undeservingly worse off compared others (Smith et al., 2012; Van den Bos, 2018). Unlike objective deprivation, which captures more tangible indicators, such as poverty or low educational attainment, it is the subjective perception and related experience of deprivation in comparison to other groups which matter (Power, 2018). While objective deprivation may be present at the same time and likely influences subjective feelings of deprivation (Jetten et al., 2020), it needs to be perceived as unjust in order to evoke group-based emotions and behavioral intentions (Jetten et al., 2017). This is in line with a recent meta-analysis which found that different measures of objective deprivation (e.g., SES, unemployment, level of education, and income) are weak and often non-significant predictors for different cognitive and behavioral radicalization outcomes (Wolfowicz et al., 2020). Yet, small to medium sized effects emerged for group-based relative deprivation in predicting extremist attitudes and behavioral intentions (Ibid).

For decades, relative deprivation has been a prominent explanation why individuals engage in social and political protest behavior (for a meta-analytic review, see Smith et al., 2012). Research on collective action has provided an extensive empirical evidence base on the relationship between relative deprivation, including negative group-based emotions such as feelings of injustice and anger, strong group identification and engagement on behalf of a group to redress the perceived injustice (e.g., Simon and Klandermans, 2001; Walker and Smith, 2002; Van Zomeren et al., 2008; Abrams and Grant, 2012). Relatedly, research on violent extremism demonstrates that a crystallization of perceived injustices and feelings of discrimination may explain why individuals adopt extremist propensities and engage in extremist violence (Agnew, 2016).

For instance, large scale studies among German, Belgian and Dutch majority members highlight several direct and indirect effects between perceived injustices and relative deprivation and individuals’ right-wing violent extremist attitudes, intentions (Doosje et al., 2012) and behavior (Boehnke et al., 1998). Doosje et al. (2013) found similar results among a sample of Dutch Muslim youth, whereby perceived injustices were associated with the adoption of a radical belief system and support for extremist violence. In addition, several structural equation models highlight that perceived group deprivation and injustices seem to trigger the onset of other risk factors associated with violent extremist intentions (Rottweiler et al., 2020), self-reported political violence (Pauwels et al., 2018) and self-reported right-wing extremist violence (Pauwels and Heylen, 2020). Further evidence on the relative deprivation and violent extremism link was provided by Obaidi et al. (2019). Across several studies, Obaidi et al.’s (2019) findings demonstrated that perceptions of group injustice are significantly related to different extremism outcomes among Western-born Muslims, thus rendering it a fundamental factor in understanding support for extremism. Additionally, perceived injustice demonstrated an indirect positive effect on violent intentions via group-based anger among Danish Muslims (Obaidi et al., 2018) and among Muslims living in Western countries as well as Muslims in Afghanistan and Pakistan (Obaidi et al., 2020).

Collectively, these results suggest that group-based relative deprivation and associated feelings of perceived injustice may predict increased support for and willingness to engage in violent extremism. However, it is important to emphasize that relative deprivation does not necessarily lead to radicalization. In fact, research shows that only some of those who experience such strains develop extremist beliefs (Kruglanski and Fishman, 2009; Sageman, 2014; Agnew, 2016; Rottweiler et al., 2020). To account for this, individual differences as potential moderators are worthy of consideration (e.g., Borum, 2014; McGregor et al., 2015). Individual differences in personality affect the way in which individuals react to environmental and situational stressors, rendering perceptions, behavioral intentions as well as actual behavior dependent on the interplay between these factors (e.g., Mondak, 2010; Gallego and Oberski, 2012).

While individual and contextual factors may independently influence individuals’ risk of radicalization, their interactions may exert particularly strong effects (Ozer and Bertelsen, 2019). Such an emphasis on the dynamic interplay between individual differences and contextual factors within radicalization processes has become prominent within psychological theories of violent extremism (e.g., Doosje et al., 2016; Gøtzsche-Astrup, 2018). For instance, Gøtzsche-Astrup’s (2019) survey studies found significant interactive effects between different personality traits and contextual factors, such as uncertainty, on violent extremist intentions. Ozer et al. (2020) showed that different aspects of one’s social identity moderated the effects of insecure life attachment on different extremist measures. Similarly, Pavlović and Franc’s (2021) results highlighted significant interaction effects among individual dispositions and perceptions of contextual factors. The findings demonstrated that dark personality traits moderate the effects of relative group deprivation on support for political violence and radical intentions. While Pavlović and Franc (2021) provide evidence for the conditional risk effects of subjective deprivation and Dark Tetrad traits, no interactive protective or buffering effects were studied. Yet, certain individual differences may increase or conversely may dampen the adverse effects of contextual circumstances upon the endorsement of, and intentions to engage in, extremist violence. The impact of individual differences upon group-based relative deprivation and subsequent perceptions of injustice remains largely unexplored, however.

## Present Study

Overall, we still know very little about the interactional and contextual effects as well as the functional relevance of certain risk and protective factors for radicalization and violent extremism (Gill, 2015). Therefore, the present study begins to delineate some of these risk and protective factor relationships. We assess the relationship between group-based relative deprivation and support for as well as willingness to engage in extremist violence using a United Kingdom nationally representative sample (by age, gender, and ethnicity). The analyses examine whether this relationship is contingent on individual differences in personality. We expect to find significant person-contextual interactions. More specifically, we expect significant interactive effects between perceptions of contextual factors, such group-based relative deprivation and several individual differences on violent extremist attitudes and intentions.

This paper comprises of two sets of analyses stemming from the same dataset. Study 1A analyses risk × risk interactions and estimates whether the relationship between relative deprivation and violent extremist *attitudes* and violent extremist *intentions* is dependent on individuals’ levels of needs for uniqueness and status as well as varying levels of trait entitlement. More specifically, we examine whether the identified risk factors will interact with each other, whereby particularly the co-occurrence of these factors is assumed to significantly increase the risk for violent extremist attitudes and intentions. Whereas Study 1A examines risk × risk interactions, Study 1B focuses on risk × protective interactions. Thus, the second set of analyses test whether the relationship between relative group deprivation and violent extremism is contingent on various protective factors being present. We examine whether potential protective factors, i.e., trait forgiveness, high levels of self-control as well as critical- and open-minded thinking styles may dampen or nullify the adverse effects of group deprivation on violent extremism. The following sections provide the rational for selecting the potential risk and protective factor moderators.

## Study 1A

### Need for Uniqueness

The need for uniqueness is a stable personality trait which denotes a need or desire to be different from others (Lynn and Snyder, 2002). People’s need for uniqueness and their desire to be special have been described as fundamental human motives (Gebauer et al., 2014). This assumption aligns with the significance quest theory of radicalization (SQT; Kruglanski et al., 2014). SQT emphasizes the contribution to radicalization outcomes made by one’s aim to achieve significance and uniqueness (Kruglanski and Webber, 2014). Endorsement of extremist ideologies and engagement in extremist groups have been argued to meet basic psychological needs (Jasko et al., 2017). Research suggests individuals adopt and accept extremist attitudes due to identity needs, pertaining to feelings of uniqueness, belonging as well as a need for certainty (Kenig, 2019). The adoption of particular beliefs is thought to fulfill such a need for uniqueness (Fromkin and Snyder, 1980). Thus, a strong need for uniqueness may be relevant for understanding individuals’ attraction and involvement within fringe movements. In a similar way to why many individuals are drawn toward conspiracy theories (e.g., to fulfill certain psychological needs, such as feeling special and unique), the endorsement of extremist ideologies and engagement in extremist groups have been argued to meet these needs (Jasko et al., 2017; Sternisko et al., 2020). Hence, individuals who exhibit a strong motivation to be unique and different may be particularly prone to hold extremist beliefs and to engage in non-normative political action (Sternisko et al., 2020).

### Need for Status

Empirical studies also suggest the need for status as a potential risk factor for engagement in violent extremism by increasing individuals’ susceptibility and attraction to extremist groups. For instance, joining violent extremist groups and adopting extremist ideologies have been argued to offer individuals a sense of fulfillment and status (Sageman, 2011). Relatedly, status seeking has been described as a basic social-psychological factor fundamental to extremist radicalization and recruitment processes (Dandurand, 2015). The Extremism Risk Guidelines (ERG22+), which is a SPJ guidance for the risk assessment of violent extremists, lists the ‘need for status’ as a risk factor that may increase individuals’ identification and engagement with an extremist ideology and/or group (Powis et al., 2019).

Frustrated status needs are one of the factors which draw mainly young men toward involvement within criminal gangs and extremist groups in order to restore or enhance social status and to attain self-esteem (Silke, 2008; Bartlett et al., 2010). Venhaus’ (2010) report on over 2,000 interviews and histories of foreign fighters identifies status seeking as a way to achieve recognition and a key factor why young men join terrorist groups. Similarly, SQT highlights individual motivations driving radicalization processes, whereby personal significance, including a sense of recognition and status, represent fundamental human needs that can be achieved or restored by engaging in extreme behavior (Kruglanski et al., 2014). The need to achieve status and significance underlie the desire to matter and to be recognized (Webber et al., 2018). Thus, adopting an extremist ideology may meet individuals’ need for existential meaning, by providing a clear purpose, such as achieving status and respect within groups (Horgan, 2008; MacDougall et al., 2018).

### Trait Entitlement

Trait entitlement has varyingly been considered as either a sub-trait of narcissism (Miller et al., 2012) or a relatively independent construct. It refers to a stable personality characteristic that one is more deserving and entitled to more compared to other people (Campbell et al., 2004). More specifically, it captures rigid beliefs relating to feelings of inflated deservingness, perceptions of being special and privileged, alongside exaggerated expectations and exploitative tendencies (Moeller et al., 2009; Grubbs and Exline, 2016). Trait entitlement influences individuals’ attitudes, intentions and behaviors across situations. For instance, previous research confirms a significant relationship between entitlement and hostility, extreme aggression and violence perpetration (Ruiz et al., 2001; Reidy et al., 2008; Burt et al., 2012).

In addition, findings highlighted that narcissistic entitlement is the narcissistic sub-trait that most strongly predicts different measures of aggression (Reidy et al., 2008). In fact, entitlement and exploitativeness emerged as the only significant predictors of aggression when all narcissism sub-traits were entered simultaneously in the regression model, thus reflecting an extreme maladaptive trait of narcissism. Bushman et al. (1999, cited in Baumeister et al., 2000) found similar results among incarcerated violent offenders who demonstrated significantly increased levels of entitlement. Relatedly, inflated feelings of superiority and a strong sense of entitlement to special privileges constitute particularly relevant risk factors for aggression and violent behavior (Baumeister et al., 2000).

Furthermore, trait entitlement showed a positive relationship with different measures of aggression via feelings of perceived injustice (Archer and Thanzami, 2009). Unmet expectations violate entitled individuals’ notions of deservingness. In the wake of such violated expectations, individuals high in entitlement are more likely to interpret the event as a perceived injustice (Grubbs and Exline, 2016). Like other maladaptive personality characteristics, trait entitlement can lead to increased and continual vulnerability due to constant unmet expectations as well as entitled interpretations and distressing reactions toward those, fostering perceptions of injustice and unfair treatment (Twenge and Campbell, 2003; Miller et al., 2009). Such a propensity for frequently violated expectations renders highly entitled people particularly prone to engage in anger rumination and revenge planning, which ultimately increases the risk toward violence to pursue ‘justice’ (Raskin and Novacek, 1991; Grubbs and Exline, 2016).

#### Hypotheses

First, based on findings from the literature review, we run several moderation analyses, which will be detailed in the following. We expect group-based relative deprivation to be significantly and positively related to support for and intentions to engage in extremist violence. In addition, we examine how several individual differences (e.g., trait entitlement, need for status, and need for uniqueness) can moderate the relationship between group-based relative deprivation and violent extremist attitudes and violent extremist intentions.

We expect that the relationship between group deprivation and violent extremist attitudes (H1) and violent extremist intentions (H2) will be moderated by uniqueness needs. More specifically, we expect that individuals with a strong disposition toward uniqueness and who hold stronger feelings of relative deprivation, will show the strongest support for and readiness to engage in extremist violence.

We further expect that the relationship between relative group deprivation and violent extremist attitudes (H3) and violent extremist intentions (H4) will be moderated by status needs. We expect that those individuals who score high in need for status and experience strong group injustice, will hold an increased risk of support for and readiness to engage in violent extremism. Individuals who hold perceptions of group deprivation may be more likely to engage in violent extremism when they additionally hold strong status needs. This may be due to the fact that people who experience injustice are unlikely to have their status needs fulfilled. Engagement in extremist groups and behavior may provide an opportunity to regain status and redress injustices.

Lastly, we expect that the effects of group-based relative deprivation on violent extremist attitudes (H5) and violent extremist intentions (H6) will be moderated by levels of entitlement.

### Method

#### Participants

The data collection took place in July 2020. Participants were recruited via Prolific. Participants were based on a United Kingdom nationally representative sample (by age, gender, and ethnicity) *n* = 1,500. Overall, 51.3% (*n* = 769) identified as female, 48.7% (*n* = 730) identified as male and one individual indicated non-binary as their gender status (*M*_age_ = 44.92; *SD*_age_ = 15.91). The majority of participants (*n* = 1275; 85%) stated ‘White’ as their ethnicity. This was followed by 7.7% (*n* = 115) who stated ‘Asian,’ 3.7% (*n* = 55) identified as ‘Black.’ In total, 2% of respondents (*n* = 31) indicated ‘Mixed’ as well as 1.6% (*n* = 24) answered ‘Other.’ Education levels varied across participants: 2% had no formal qualifications, 17.8% of participants had GCSEs (or equivalent), 24.5% had A-levels/BTEC, 38% held an undergraduate degree, 13.8% held a Masters degree, and 2.9% of all participants completed a Ph.D.

#### Procedure

Participants were invited to participate in a study on risk and protective factors for violent extremism. After completing the consent form, participants were asked to fill out the questionnaire. Upon completion of the questionnaire, the respondents were thanked and debriefed. Participants received a small participation fee. After the data collection finished, the data was examined to ensure data quality and to check for any missing data. We further reviewed whether respondents had missed attention checks and we assessed the completion time for each participant. Participants were excluded from the data analysis if they missed more than two attention checks and when they completed the survey more than two standard deviations quicker than the average completion time.

#### Measures

Throughout both studies, all items were measured on 7-point scales from 1 (*strongly disagree*) to 7 (*strongly agree*). For all scales, the individual scale items were averaged into a score for each respondent, whereby higher values denoted: stronger support for violent extremism, a greater willingness to engage in violent extremism, higher levels of perceived group deprivation, higher levels of trait entitlement, a stronger need for uniqueness, and a stronger need for status.

##### Violent Extremist Attitudes

The violent extremist attitudes scale is a four-item measure of generic support for violent extremism, which has been developed for the Zurich Project on the Social Development of Children and Youths (z-Proso), an ongoing prospective longitudinal study on the development of aggressive and other problem behavior (e.g., ‘*It’s OK to support groups that use violence to fight injustices*,’ ‘*It’s sometimes necessary to use violence, commit attacks, or kidnap people to fight for a better world*,’ ω = 0.88, Nivette et al., 2017).

##### Violent Extremist Intentions

We assessed individuals’ violent extremist intentions with four items from the Radicalism Intention Scale (RIS), which is a validated and widely used scale to measure participants’ willingness to engage in different illegal and violent behaviors on behalf of a group (e.g., ‘*I would participate in a public protest against oppression of my group even if I thought the protest might turn violent*,’ ‘*I would attack police forces if I saw them beating members of my group*,’ ω = 0.84; Moskalenko and McCauley, 2009).

##### Group Injustice

The present conceptualization of group-based relative deprivation entails a (1) cognitive component, such as thoughts that one’s group receives less than one feels rightfully entitled to and is relatively disadvantaged over other groups and an (2) affective component, such as feelings of anger over this injustice (e.g., Smith and Pettigrew, 2015). Four items measured the construct of group-based deprivation, i.e., perceived injustice, discrimination and unfair treatment felt on behalf of the group the participant most strongly identified with (e.g., ‘*It makes me angry when I think of how my group is treated in comparison to other groups in the United Kingdom*’ and ‘*If I compare the group to which I belong with other groups in the United Kingdom, I think we are treated unfairly*’ (ω = 0.93). The items were originally developed for a Dutch survey measuring attitudes toward extremism conducted by Van den Bos et al. (2010) and have afterward been translated into English by Pauwels and De Waele (2014).

##### Trait Entitlement

We operationalized the psychological entitlement scale (PES) by Campbell et al. (2004) to capture individuals’ inflated notions of deservingness and entitlement regarding the self (e.g., *‘I honestly feel I’m just more deserving than others,’ ‘I feel entitled to more of everything,’*ω = 0.89). Across several studies, the PES has demonstrated good psychometric properties. It has shown to be a reliable and valid measure and to be stable across time (Ibid).

##### Need for Status

Dispositional need for status was assessed with the affiliation motivation scale, which measures individuals’ desire to attain status, recognition, and respect from others (e.g., ‘*I mainly like to be around others who think I am an important, exciting person*,’ ‘*I often have a strong desire to get people I am around to notice me and to appreciate what I am like*,’ ω = 0.91, Hill, 1987).

##### Need for Uniqueness

The need for uniqueness was assessed with the 4-item Self-attributed Need for Uniqueness scale (SANU; Lynn and Snyder, 2002), which measures individuals’ self-reported desire to be different from others (e.g., ‘*Being distinctive is important to me*,’ ‘*I have a strong need for uniqueness*,’ ω = 0.89).

#### Statistical Analysis

We ran a series of moderation analyses to examine the expected interactive effects of group deprivation and several individual differences in personality on violent extremism. We estimated all our interaction models in the software program R using the packages ‘jtools’ (Long, 2020a) and ‘interactions’ (Long, 2020b). We created average scores of our scales which were entered into the regression models. We calculated robust standard errors to apply a heteroskedasticity-consistent standard error estimator and to handle the violation of the normality assumption of our dependent variable (Zeileis et al., 2019). In addition, we applied a mean centering technique to all our continuous independent variables to yield interpretable coefficients (Aiken et al., 1991; Hayes, 2017). Probing and plotting of the interaction models were conducted in R with the function ‘probe_interaction,’ which combines the functions ‘sim_slopes’ and ‘interaction_plot’ (Long, 2020b). We controlled for age, gender, and more objective measures of deprivation, such as level of education and family income within all models due to the potential relationship with violent extremism (the analyses without the covariates yielded almost the same results). The models were run with 5,000 bootstrap samples and 95% bias corrected bootstrap confidence intervals as this method is robust to non-parametric data and statistical outliers and effectively handles deviations from the normal distribution of study variables as no assumptions about the shape of the sampling distribution are made (Preacher et al., 2007). We ran all moderation models with two different operationalizations of violent extremism, i.e., violent extremist *attitudes* and violent extremist *intentions* to increase the generalizability and validity of our study findings and thus, to increase the robustness of findings. This further allowed us to examine whether the effects differed depending on the operationalization of violent extremism (e.g., whether there are differences predicting violent extremist attitudes compared to violent extremist intentions).

## Results

The CFAs on all scale measures were run. All indicators showed satisfactory factor loadings with standardized coefficients ranging from β = 0.62 to β = 0.91. Table 1 displays the correlations among all variables. All independent variables were positively and significantly correlated with violent extremist attitudes and violent extremist intentions.

**TABLE 1:** Descriptive statistics and correlations among variables of interest, Study 1A.

Variables	Correlations						
	*M* (*SD*)	1	2	3	4	5	
(1) Violent extremist attitudes	2.37 (1.38)	–					
(2) Violent extremist intentions	2.68 (1.29)	0.62***	–				
(3) Group deprivation	3.06 (1.49)	0.23***	0.30***	–			
(4) Need for uniqueness	3.84 (1.28)	0.16***	0.25***	0.17***	–		
(5) Need for status	2.83 (1.28)	0.18***	0.26***	0.18***	0.34***	–	
(6) Trait entitlement	2.58 (1.18)	0.13***	0.15***	0.36***	0.21***	0.06*	–

Pearson’s correlation coefficients are reported. n = 1,500. *p < 0.05 and ***p < 0.001.

To test our risk × risk hypotheses, we ran a series of moderation analyses, with group-based deprivation as the independent variable, need for uniqueness, need for status and trait entitlement as moderating variables, and violent extremist attitudes and violent extremist intentions as the outcome variables.

The results from our first analysis confirm that group-based relative deprivation is positively associated with violent extremist attitudes (*b* = 0.21, 95% CI [0.17, 0.26]) and violent extremist intentions (*b* = 0.26, 95% CI [0.21, 0.30]). This finding indicates that individuals who hold stronger perceptions of group-based injustice hold higher levels of support for violent extremism and exhibit a stronger willingness to engage in violent extremism.

For the interaction analyses’ first two models, the centered main effects showed that both relative group deprivation (*b*_Attitudes_ = 0.19, 95% CI [0.15, 0.24]; *b*_Intentions_ = 0.23, 95% CI [0.18, 0.27]) and need for uniqueness (*b*_Attitudes_ = 0.14, 95% CI [0.08, 0.19]; *b*_Intentions_ = 0.21, 95% CI [0.17, 0.26]) were positive and significant predictors of violent extremist attitudes and violent extremist intentions. In line with our predictions, need for uniqueness significantly moderated the effects of relative deprivation on violent extremist attitudes (H1; *b* = 0.06, 95% CI [0.03, 0.09]) and violent extremist intentions (H2; *b* = 0.04, 95% CI [0.01, 0.07]). These results confirm that the effects of group deprivation and both violent extremism outcomes are conditional on individuals’ uniqueness needs.

**FIGURE 1:**
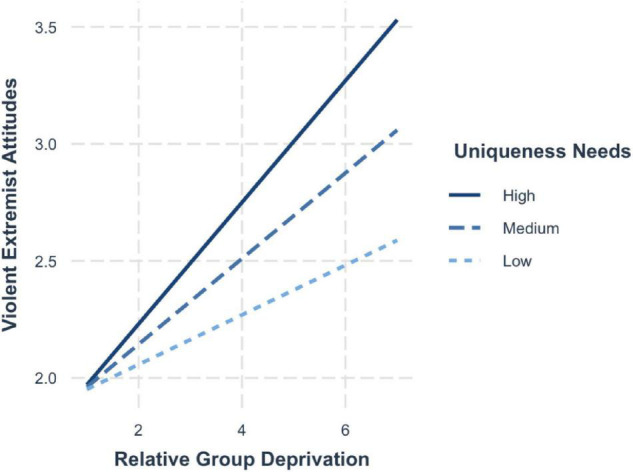
Simple slopes for group deprivation and need for uniqueness on violent extremist attitudes.

To illustrate the significant interactions of relative deprivation and need for uniqueness, we computed simple slopes. The plotted values of the predictors represent one standard deviation above (+1 SD; high), at the mean (average) and one standard deviation below (−1 SD; low) the mean using the procedures outlined by Aiken et al. (1991). The probing of the conditional effects at different levels of the moderator shows that when perceptions of group deprivation are strong, high uniqueness needs exert strong positive effects on violent extremist attitudes (+1 SD; *b* = 0.26, 95% CI [0.20, 0.32]) (Figure 1). These effects are attenuated when the need for uniqueness is average (mean; *b* = 0.20, 95% CI [0.16, 0.25]) and further weakened when uniqueness needs are low (−1SD; *b* = 0.11, 95% CI [0.05, 0.18]).

**FIGURE 2:**
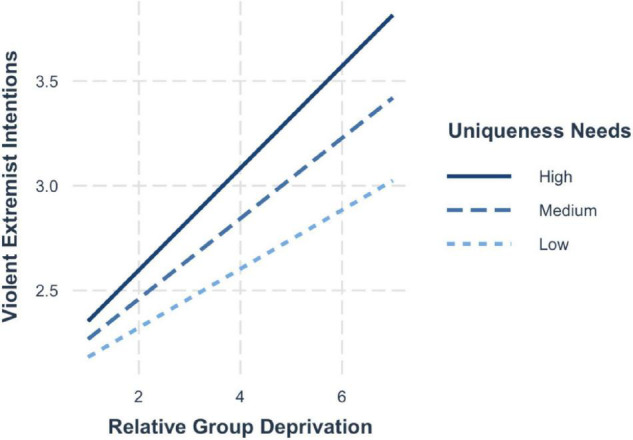
Simple slopes for group deprivation and need for uniqueness on violent extremist intentions.

Similar results emerged for violent extremist intentions, whereby the risk effects were strongest when in addition to high group deprivation, individuals also held a high disposition for uniqueness (+1 SD; *b* = 0.27, 95% CI [0.22, 0.32]). The effects are lower for average levels of uniqueness needs (mean; *b* = 0.23, 95% CI [0.19, 0.27]) and the lowest when the need for uniqueness was low (−1 SD; *b* = 0.17, 95% CI [0.12, 0.23]) (see Figure 2).

The findings from model 3 and model 4 revealed that when relative group deprivation (*b*_Attitudes_ = 0.18, 95% CI [0.14, 0.23]; *b*_Intentions_ = 0.22, 95% CI [0.18, 0.26]) and need for status (*b*_Attitudes_ = 0.15, 95% CI [0.10, 0.21]; *b*_Intentions_ = 0.21, 95% CI [0.16, 0.26]) were entered simultaneously into the regression, both showed a positive and significant association with violent extremist attitudes and violent extremist intentions. In addition, need for status had a significant moderating effect on the relationship between relative group deprivation and violent extremist attitudes (H3; *b* = 0.04, 95% CI [0.01, 0.07]). Yet, contrary to what we expected, need for status did not moderate the relationship between group deprivation and violent extremist intentions (H4; *b* = 0.03, 95% CI [−0.004, 0.05]).

**FIGURE 3:**
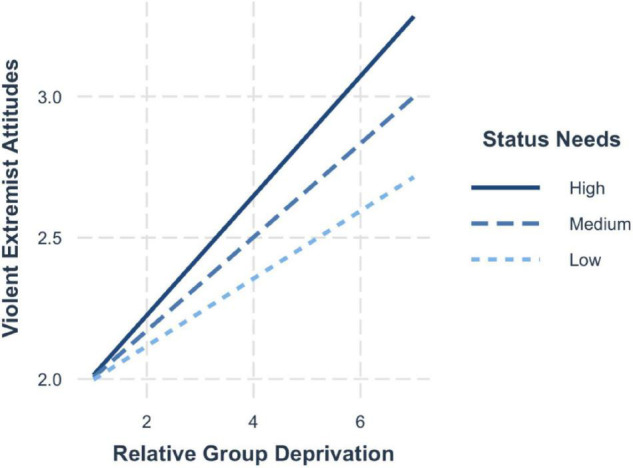
Simple slopes for group deprivation and need for status on violent extremist attitudes.

Simple slopes (Figure 3) illustrate that the effects of group deprivation on violent extremist attitudes are particularly strong among those high in status needs (+ 1SD; *b* = 0.23, 95% CI [0.17, 0.30]). The probing of the interaction reveals that the effects are lessened for those scoring average on the need for status (mean; *b* = 0.18, 95% CI [0.13, 0.22]) and lowest among those who exhibit low status needs (−1 SD; *b* = 0.13, 95% CI [0.07, 0.19]).

Model 5 showed that both group-based relative deprivation (*b*_Attitudes_ = 0.20, 95% CI [0.15, 0.25]) but not trait entitlement (*b*_Attitudes_ = 0.05, 95% CI [−0.01, 0.11]) significantly predicted violent extremist attitudes. The findings from model 6 found group deprivation (*b*_Intentions_ = 0.24, 95% CI [0.19, 0.28]) and trait entitlement (*b*_Intentions_ = 0.06, 95% CI [0.01, 0.12]) to be significant and positive predictors of violent extremist intentions when entered together into the regression equation. In line with our expectations, a significant interaction between group deprivation and trait entitlement emerged, whereby the relationship between group deprivation and violent extremist attitudes was moderated by trait entitlement (H5; *b* = 0.05, 95% CI [0.01, 0.08]).

**FIGURE 4:**
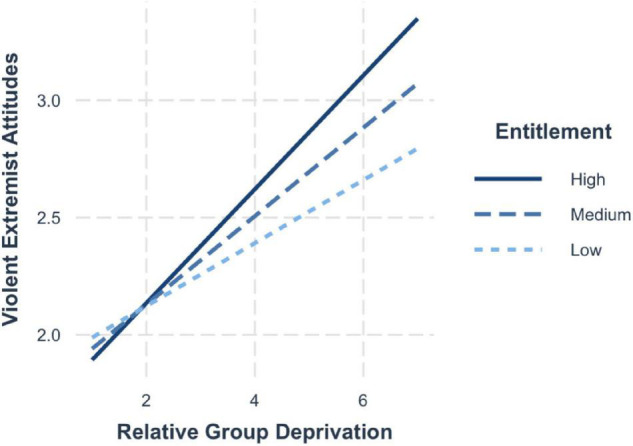
Simple slopes for group deprivation and trait entitlement on violent extremist attitudes.

The probing of the conditional effects showed that when trait entitlement is high, the effects of group deprivation are amplified (+1 SD; *b* = 0.26, 95% CI [0.19, 0.33]). The effects were dampened when levels of entitlement were average (mean; *b* = 0.18, 95% CI [0.13, 0.23]) and lowest when entitlement was low (−1 SD; *b* = 0.14, 95% CI [0.07, 0.20]) (Figure 4).

Contrary to what we expected, the interaction between group deprivation and trait entitlement proved to be non-significant for violent extremist intentions (H6; *b* = 0.01, 95% CI [−0.02, 0.04]). Thus, trait entitlement did not moderate the effects of group deprivation on violent extremist intentions.

## Study 1B

Study 1B examines whether certain factors may exert protective factors against support for and intentions to engage in extremist violence for individuals who experience relative group deprivation.

### Trait Forgiveness

Trait forgivingness denotes a disposition to forgive interpersonal transgressions over time and across situations (Berry et al., 2005). Relatedly, forgiveness has been described as the ability to let go of negative emotions, vengeful feelings and resentment related to a perceived transgression and describes a way of adaptive responding following suffering (Exline et al., 2003; McCullough et al., 2007). Forgiveness is further seen as a way to restore interpersonal and intergroup harmony after transgression (McCullough et al., 2000; Worthington, 2007). Various studies analyzed the correlates of trait forgiveness. For instance, trait forgivingness was negatively associated with chronic hostility, trait anger and vengeful rumination (Berry et al., 2001). It was further positively related to several traits linked to positive and pro-social affect, such as empathic concern and empathic perspective taking (Ibid) as well as agreeableness (Worthington and Wade, 1999). Conversely, unforgiveness has been described as a process whereby people hold on to negative emotions, bolstering a sense of victimhood (Wade and Worthington, 2005). This corresponds with the concept of revengefulness which denotes a tendency to insist on revenge and thus, relates to the inability to forgive perceived insults or harms (Wade et al., 2008).

### Self-Control

Gottfredson and Hirschi (1990) argue that the ability to execute self-control is a key factor in explaining delinquency and the development of criminal propensities. Gottfredson and Hirschi originally conceptualized six dimensions of self-control: risk-taking behavior, immediate gratification, preference for simple tasks, volatile temper, impulsiveness, and self-centeredness. More recently, quantitative research extended this link to the explanation of violent extremism with a predominant focus on the aspect of thrill-seeking, risk-taking and impulsivity (Grasmick et al., 1993; Pauwels and De Waele, 2014; Rottweiler and Gill, 2020). Survey studies corroborate that a poor ability to execute self-control is significantly correlated with exposure to extremist settings and self-reported violent extremist attitudes and behavior, irrespective of ideology (Pauwels and Hardyns, 2018; Rottweiler et al., 2020; Schumpe et al., 2020). Qualitative research analyzing right-wing extremist groups, also highlighted the importance of thrill-seeking and risk-taking as key determinants in explaining involvement in extremism and violence committed by far-right extremists (see for example Bjørgo, 2002; Bouhana et al., 2018; Lakhani and Hardie-Bick, 2020). These findings suggest that the receptivity to extremist ideologies is associated with poor self-regulation (Bouhana, 2019).

### Critical Thinking

A prominent theme within the prevention of violent extremism is to strengthen resilience within individuals. One such preventative approach focusses on developing cognitive resources and to help individuals to become critical as well as flexible in their thinking. By developing and strengthening certain cognitive skills and capacities, individuals are thought to be better equipped to critically assess and question extremist propaganda which, in turn, increases resistance toward the attraction of such messages (Stephens et al., 2021). Yet, rather than focusing on the extremist messages themselves, the way individuals think and process information is seen as crucial for preventing extreme and simplistic categorizations, often labeled as black-and-white-thinking in which narratives such as ‘us versus them’ or ‘good and evil’ may become embedded (Liht and Savage, 2013). As such, a promising pathway for interventions is to increase cognitive complexity and to particularly strengthen critical thinking capabilities. Enhancing critical thinking may act as a protective factor against violent extremism by strengthening the ability to critically engage with information and messages as well as to critically assess and question the source and content of ideas, which ultimately may build resilience against the attraction of extremist ideas and groups (Davies, 2009; Mattsson and Säljö, 2018).

#### Hypotheses

The above accounts suggest that various protective factors may dampen the effects of risk factors for violent extremism. Based on research outlined in the literature review, we run several interaction models (see below). We examine how several individual differences (e.g., trait forgiveness, the ability to execute self-control, and critical thinking dispositions) may moderate the relationship between group-based relative deprivation and violent extremism.

We expect that trait forgiveness will moderate the effects of perceived group injustice on violent extremist attitudes (H1) and violent extremist intentions (H2), whereby higher levels of trait forgiveness will lessen the risk effects.

We expect that self-control will moderate the effects of perceived group injustice on violent extremist attitudes (H3) and violent extremist intentions (H4), whereby higher levels of self-control will lessen the risk effects.

We expect that critical thinking will moderate the effects of perceived group injustice on violent extremist attitudes (H5) and violent extremist intentions (H6), whereby higher levels of critical thinking will lessen the risk effects.

### Method

#### Participants and Procedure

Participants were part of the same sample used in Study 1A and the same dataset was used to estimate the models in Study 1B. Data collection and cleaning procedures have already been outlined above.

#### Statistical Analysis

In Study 1B, we ran several interaction models to examine the expected interactive protective effects of group-based relative deprivation and several individual differences on violent extremism. The statistical procedures are the same as the ones detailed in Study 1. Like in the previous study, we controlled for age, gender, and more objective measures of deprivation, such as level of education and family income within all models (the analyses without the covariates yielded very similar results). As for Study 1A, the models were run with 5,000 bootstrap samples and 95% bias corrected bootstrap confidence intervals to account for the non-normal distribution of the outcome variables. We ran all moderation models with two different operationalizations of violent extremism (e.g., violent extremist *attitudes* and violent extremist *intentions*).

#### Measures

Violent extremist attitudes, violent extremist intentions and group-based deprivation are described in Study 1A. Like in the previous study, all items were measured on a 7-point Likert scale ranging from 1 (*strongly disagree*) to 7 (*strongly agree*). The individual scale items were averaged to calculate a score for each participant, whereby higher scores indicated, e.g., higher levels of trait forgiveness, a higher self-reported critical thinking disposition and a strong ability to execute self-control.

##### Trait Forgiveness

The validated 10-item ‘Trait Forgiveness Scale’ (Berry et al., 2005) was operationalized. Trait forgiveness refers to the disposition to forgive interpersonal transgressions over time and across situations (e.g., ‘*I can usually forgive and forget an insult*,’ ‘*I have always forgiven those who have hurt me*,’ ω = 0.81). The trait forgiveness scale demonstrated construct validity and empirical concurrent validity. The scale showed positive correlations with other validated dispositional forgiveness scales and was found to be negatively associated with trait anger, hostility, aggression, and vengeful rumination and was further positively correlated with agreeableness and empathy (Berry et al., 2005).

##### Self-Control

To assess participants’ self-reported ability to exercise self-control, we measured a modified 7-item version of the self-control scale developed by Grasmick et al. (1993), which taps into the concepts of thrill-seeking, impulsivity and risk-taking (e.g., ‘*When I am really angry, other people better stay away from me*,’ ‘*Sometimes I find it exciting to do things that may be dangerous*,’ ω = 0.84).

##### Critical Thinking Disposition Scale

Critical thinking was measured with the ‘Critical Thinking Disposition Scale’ (CTDS) (Sosu, 2013). The scale is comprised of two subscales, ‘Critical Openness’ and ‘Reflective Skepticism’ (e.g., ‘*It’s important to understand other people’s viewpoint on an issue,’* ‘*I often think about my actions to see whether I could improve them*,’ ω = 0.85). The critical openness subscale describes individuals’ tendencies to be actively open to new ideas, but also to be critical in evaluating those and further captures the disposition to modify one’s thinking when faced with new and convincing evidence. The reflective skepticism subscale refers to the tendency to learn from past experiences and to question evidence before making decisions (Sosu, 2013).

## Results

The CFAs were conducted for all additional measures, which had not been operationalized in the previous study, i.e., trait forgiveness, self-control and critical thinking. All indicators showed satisfactory factor loadings with standardized coefficients ranging from β = 0.59 to β = 0.93. Table 2 displays the correlations among all variables operationalized within Study 1B. Trait forgiveness, self- control and critical thinking showed significant positive correlations among each other, and they were negatively and significantly correlated with relative group deprivation, violent extremist attitudes and violent extremist intentions.

**TABLE 2:** Descriptive statistics and correlations among variables of interest, Study 1B.

Variables 2		Correlations					
	*M* (*SD*)	1	2	3	4	5	6
(1) Violent extremist attitudes	2.37 (1.38)	–					
(2) Violent extremist intentions	2.68 (1.29)	0.62***	–				
(3) Group deprivation	3.06 (1.49)	0.23***	0.30***	–			
(4) Trait forgiveness	4.57 (1.01)	−0.22***	−0.25***	−0.23***	–		
(5) Self-control	5.18 (1.41)	−0.30***	−0.34***	−0.20***	−0.42***	−	
(6) Critical thinking	5.46 (0.73)	−0.11***	−0.15***	−0.14***	−0.22***	−0.21***	–

Pearson’s correlation coefficients are reported. n = 1500. ***p < 0.001.

Within model 1 and model 2, the centered main effects demonstrated that group relative deprivation (*b*_Attitudes_ = 0.17, 95% CI [0.13, 0.22]; *b*_Intentions_ = 0.21, 95% CI [0.17, 0.26]) is a positive and trait forgiveness (*b*_Attitudes_ = −0.23, 95% CI [−0.30, −0.16]; *b*_Intentions_ = −0.25, 95% CI [−0.31, −0.18]) is a negative and significant predictor for violent extremist attitudes and violent extremist intentions. In line with our first prediction, trait entitlement significantly moderated the effects of relative deprivation on violent extremist attitudes (H1; *b* = −0.05, 95% CI [−0.09, −0.01]). Contrary to our second hypothesis, no evidence was found for the moderating effects of trait forgiveness on the relationship between group deprivation and violent extremist intentions (H2; *b* = −0.01, 95% CI [−0.05, 0.03]).

**FIGURE 5:**
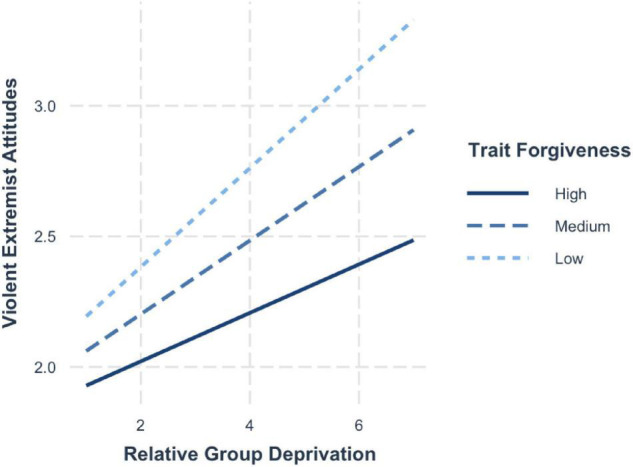
Simple slopes for group deprivation and trait forgiveness on violent extremist attitudes.

To illustrate the significant interactions of relative deprivation and trait forgiveness on violent extremist attitudes, we computed simple slopes (Figure 5). Like in the previous study, the plotted values of the predictors show the effects of one standard deviation above (+1 SD; high), at the mean (average) and one standard deviation below (−1 SD; low). The probing of the conditional effects at different levels of the moderator shows that for average (mean; *b* = 0.17, 95% CI [0.13, 0.22]) and particularly for high levels of forgiveness (+1 SD; *b* = 0.12, 95% CI [0.06, 0.19]), the risk effects of group deprivation are dampened compared to when forgiveness is low (−1 SD; *b* = 0.22, 95% CI [0.52, 1.00]).

The findings from model 3 and model 4 revealed that when group relative deprivation (*b*_Attitudes_ = 0.17, 95% CI [0.12, 0.21]; *b*_Intentions_ = 0.24, 95% CI [0.20, 0.29]) and self-control (*b*_Attitudes_ = −0.33, 95% CI [−0.39, −0.27]; *b*_Intentions_ = 0.07, 95% CI [−0.12, −0.02]) were entered simultaneously into the regression, both showed a positive and significant association with violent extremist attitudes and violent extremist intentions. In line with hypothesis 3 and 4, self-control had a significant moderating effect on the relationship between relative deprivation and violent extremist attitudes (H3; *b* = −0.05, 95% CI [−0.08, −0.01]) as well as violent extremist intentions (H4; *b* = −0.04, 95% CI [−0.07, −0.01]).

**FIGURE 6:**
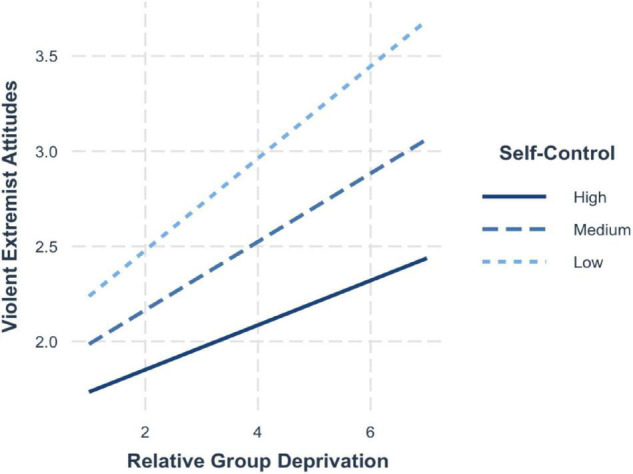
Simple slopes for group deprivation and self-control on violent extremist attitudes.

Simple slopes (Figure 6) illustrate that the effects of group deprivation on violent extremist attitudes are lessened among those high in self-control (+1 SD; *b* = 0.11, 95% CI [0.05, 0.18]) compared to those with average self-control (mean; *b* = 0.16, 95% CI [0.12, 0.21]) and particularly compared to those with low self-control (−1 SD; *b* = 0.22, 95% CI [0.15, 0.28]).

**FIGURE 7:**
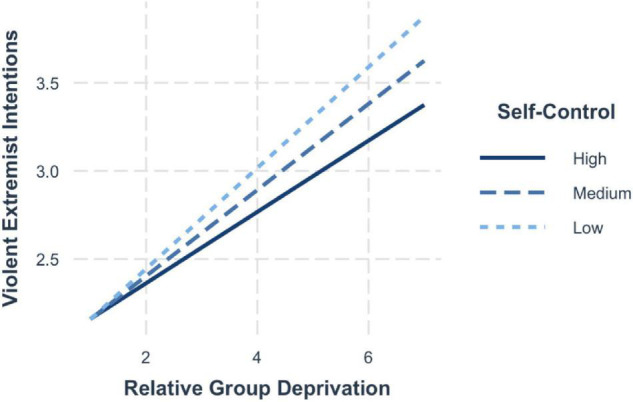
Simple slopes for group deprivation and self-control on violent extremist intentions.

Similar results emerged for the interactive effects on violent extremist intentions – for those with high self-control, strong perceptions of group deprivation still significantly increased the level of extremist intentions (+1 SD; *b* = 0.19, 95% CI [0.13, 0.26]). Yet, the effects were lessened compared to those individuals with average levels of self-control (mean; *b* = 0.24, 95% CI [0.20, 0.28]) and even weaker compared to those with low self-control (−1 SD; *b* = 0.29, 95% CI [0.23, 0.35]) (see Figure 7).

Model 5 showed that both group-based relative deprivation (*b*_Attitudes_ = 0.21, 95% CI [0.17, 0.25]) and critical thinking (*b*_Attitudes_ = −0.13, 95% CI [−0.21, −0.04]) significantly predicted violent extremist attitudes. The findings from model 6 found group deprivation (*b*_Intentions_ = 0.24, 95% CI [0.20, 0.28]) and critical thinking (*b*_Intentions_ = −0.17, 95% CI [−0.25, −0.09]) to be significant and positive predictors of violent extremist intentions when entered together into the regression equation.

**FIGURE 8:**
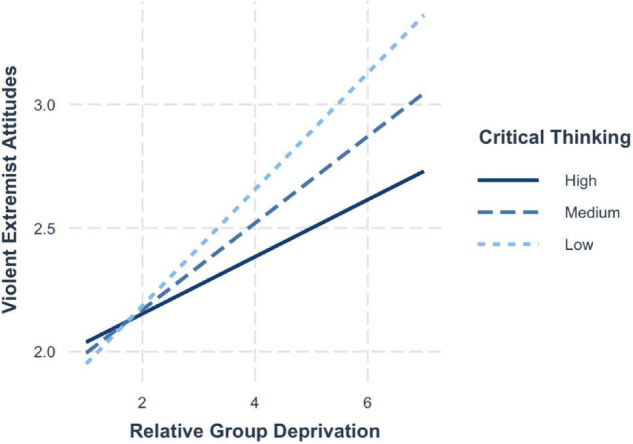
Simple slopes for group deprivation and critical thinking on violent extremist attitudes.

Confirming hypothesis 5 and 6, a significant interaction between group deprivation and trait entitlement emerged, whereby the relationship between group deprivation and violent extremist attitudes (H5; *b* = −0.08, 95% CI [−0.13, −0.02]) and violent extremist intentions (H6; *b* = −0.05, 95% CI [−0.10, −0.004]) was moderated by trait entitlement. The simple slopes (Figure 8) highlight that when critical thinking is low (−1 SD; *b* = 0.27, 95% CI [0.20, 0.33]), the risk effects of group deprivation on violent extremist attitudes are strongest. The effects are lessened when levels of critical thinking are average (mean; *b* = 0.21, 95% CI [0.16, 0.25]) and lowest when critical thinking is high (+1 SD; *b* = 0.14, 95% CI [0.08, 0.21]).

**FIGURE 9:**
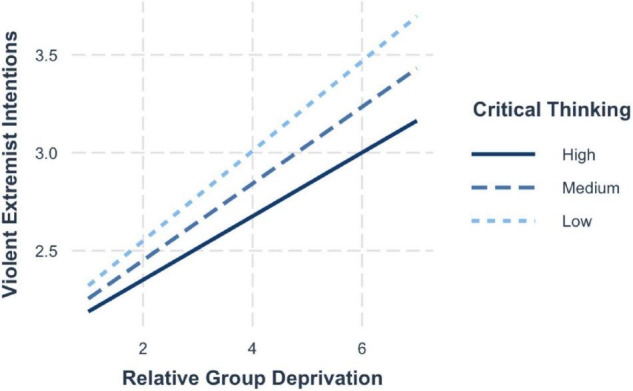
Simple slopes for group deprivation and critical thinking on violent extremist intentions.

The probing of the conditional effects showed that the effects were strongest when in addition to high group deprivation, individuals also held a low disposition for critical thinking (+1 SD; *b* = 0.21, 95% CI [0.16, 0.26]). The risk effects are attenuated for those scoring average on critical thinking (mean; *b* = 0.24, 95% CI [0.20, 0.28]) and are weakest among those who hold a strong disposition toward critical thinking (−1 SD; *b* = 0.28, 95% CI [0.22, 0.33]) (see Figure 9).

## Discussion

Our findings demonstrate that relative group deprivation predicts support for and willingness to engage in extremist violence, yet the relationship is contingent on individual differences in personality. More specifically, the results highlight various interactive effects between individual dispositions and perceptions of contextual factors, bringing us one step closer to understanding who might be more vulnerable to violent extremism as well as how.

The first set of results demonstrate that when the need for uniqueness is high, the effects of relative group deprivation on violent extremist attitudes and intentions are amplified. Similar results emerged for high status needs. The risk effects of group deprivation on support for and willingness to engage in violent extremism are strongest among those with high status needs. Thus, when uniqueness and status needs co-occur alongside perception of group deprivation, their joint influence is interactive. Individuals with high status and uniqueness needs may be particularly negatively affected by perceptions of group injustice and unfair treatment due to unmet needs for significance. Resultingly, the adoption of extremist beliefs and intentions may provide an opportunity to regain a sense of significance and to redress grievances. Therefore, it may be relevant to consider the interactive effects of status and uniqueness needs for individuals who hold strong feelings of group injustice.

Our findings further show that the effects of relative group deprivation on violent extremist attitudes are particularly strong for those individuals who also exhibit high entitlement beliefs. The effects are dampened among those with average and low levels of entitlement. Interestingly, while the interaction was significant, trait entitlement did not exert a significant main effect upon violent extremist attitudes. This indicates that instead of constituting an independent risk factor for violent extremist attitudes, entitlement seems to be only relevant in particular circumstances, for example it matters for people who experience feelings of group injustice. This is in line with previous research that found entitled people were more likely to engage in aggression against others when they experienced violated entitlement (Reidy et al., 2008). Hence, perceptions of relative group deprivation may have particularly strong effects on violent extremist beliefs among those who also hold high levels of entitlement. However, this relationship may also be spurious in that individuals may hold the view that they are deprived because of their high levels of entitlement. Therefore, their perceived injustice may simply be an entitled interpretation of unmet expectations, as individuals high in entitlement believe they have a right to those things and they also expect to receive those (Twenge and Campbell, 2003; Grubbs and Exline, 2016).

In contrast, no interactive effects between entitlement and group deprivation on violent extremist intentions were found. This is again not to say that entitlement and/or group deprivation do not matter. Both factors showed a significant positive effect on violent extremist intentions when entered simultaneously into the regression. Yet, rather than being interactive, their influence in cumulative. Within criminology that has been labeled a ‘dose-response relationship’ (Lösel and Bliesener, 2003), which indicates that adverse outcomes increase significantly as a function of accumulated risks. In such a case, more risk factors translate to more risk instead of the effects being contingent upon another. Overall, the results for the entitlement interactions confirmed that certain risk and protective factors are context-sensitive, meaning they exert differential effects within one context and as it was the case in our study, against one outcome, but may exert different effects under different conditions or for other outcomes.

Study 1B revealed several significant interactive protective effects. A buffering protective factor predicts a low probability of adverse effects of co-morbid risk factors (Rutter, 2012). For example, the effects of relative group deprivation on violent extremist attitudes and intentions were contingent upon individuals’ levels of self-control. More specifically, higher levels of self-control dampened the adverse effects of group deprivation on both violent extremism measures. These results align with previous research which found that self-control may increase resilience by exerting buffering protective effects when certain risk factors for violent extremism are present (Rottweiler and Gill, 2020). Similar findings emerged for the interaction effects between critical thinking dispositions and support for and intentions to engage in violent extremism. With higher levels of critical thinking, the adverse effects of group deprivation on violent extremism were lessened. Therefore, critical thinking acts as an interactive protective factor when perceptions of group injustice are present.

Such findings highlight that more research analyzing cognition-emotion interactions is required to examine the underlying cognitive, affective and neuropsychological mechanisms. These mechanisms are suspected to link various risk factors, including cognitive rigidity, non-critical thinking styles and poor executive functioning, such as impulsivity and risk-taking (sensation-seeking) to susceptibility to extremism (Zmigrod et al., 2021). Validated cognitive tasks that assess cognitive flexibility, executive functioning and critical thinking abilities are required (Zmigrod et al., 2021). Importantly, cognitive factors, such as critical thinking skills and cognitive flexibility may effectively reduce cognitive rigidity and enhance executive functions (Zmigrod et al., 2019) and thereby, may act as direct or interactive protective factors against developing violent extremist propensities.

Furthermore, the results showed that higher levels of trait forgiveness can buffer against the adverse effects of relative group deprivation on violent extremist attitudes but not against extremist intentions. Yet, trait forgiveness demonstrated a significant and negative main effect on violent extremist intentions, which indicates that the effects of trait entitlement and group deprivation are cumulative rather than interactive. Such an ‘inverse dose–response relationship’ may help to better understand the effects of direct protective factors, whereby the probability of adverse outcomes decreases as the number of protective factors increases (Lösel and Bender, 2003).

### Limitations

The present studies come with several limitations. First, we employed a cross-sectional research design and hence, we cannot draw any causal conclusions. While these results provide important information toward establishing an empirical evidence base on risk and protective factors for violent extremism, they cannot provide knowledge on the developmental trajectories over time. Therefore, the present cross-sectional interaction analyses represent the intermediate stage between the identification of relevant risk factors and more costly longitudinal and experimental research designs. Thus, the current results should serve to inform the selection of risk and protective factors to be included in future longitudinal research (Kraemer et al., 1997). We also acknowledge the potential limitation of the dataset as both sets of analyses were conducted with the same dataset, which might have implications in terms of the robustness of the results and we cannot be certain whether we would be able to replicate our findings within other contexts. As such, we recommend that future studies test our hypotheses within further and diverse samples.

Second, prolific is an online platform and the participant pool is limited to those individuals who sign up to the platform. Hence the sample may not be truly representative of the general population in that it is subject to selection bias. Nevertheless, Prolific affords researchers access to more novel populations than the traditional subject pool of undergraduate psychology students, and as such facilitates greater generalizability.

Third, we acknowledge shortcomings related to the operationalization of vulnerability to violent extremism. We employ proxy measures to examine individuals’ *attitudes* and *willingness* to engage in violent extremist behavior. Assessing vulnerability to radicalization is challenging, therefore attitudes as well as behavioral intentions rather than individuals’ actual behaviors were measured. Research on attitude-behavior relations suggests that under appropriate conditions, intentions can be good predictors of actual behavior (Banaji and Heiphetz, 2010; Ajzen, 2012). Criminological studies have further provided empirical evidence to support the attitudes-behavior approach arguing that criminal attitudes and intentions can lead to criminal behaviors (Folk et al., 2018).

## Conclusion

Radicalization processes and engagement in violent extremism are characterized by complex constellations of risk and protective factors (Lösel et al., 2020). This paper sought to analyze various risk-protective factor interactions for support for, and willingness to engage in, violent extremism. Our results highlight some initial empirical evidence for different interactive and cumulative effects between different risk and protective factors. We demonstrate that the effects of certain risk factors, such as relative group deprivation, are contingent on other risk and protective factors being present and thereby may lead to differential vulnerabilities to violent extremism. Importantly, the interactions between different risk and protective factors play a crucial role in predicting increased risk. Similar to other types of criminality and violence, the interactive effects of risk factors are most indicative (Lösel and Bender, 2017). Therefore, when multiple risk factors are present, rather than constituting a simple additive risk, their joint effect and interaction on the outcome variable need to be analyzed (Cicchetti et al., 1993). Therefore, future studies are required to examine the complex relationships and configurations of various risk factors which may amplify adverse effects as well as protective factors that may offset or dampen various risk effects.

Whilst our focus here was on violent extremism, potentially the same may also be true for other forms of violence and crime. The (dis)similarities between criminal and violent extremist behaviors, and those who engage in them, is certainly worthy of greater consideration. On the one hand, a recent systematic review and meta-analysis of radicalization risk factors found that the factors with the largest relative magnitude are those associated with central criminological theories (e.g., social learning, self-control, neutralization, and social control/social bonds) (Wolfowicz et al., 2020). On the other hand, some argue that violent extremism involves a different set of pro-social and affiliative motivations than common high-volume crimes (Taylor and Quayle, 1994; LaFree and Dugan, 2004; McCauley and Moskalenko, 2011).

Furthermore, our results demonstrate that to better understand why individuals are differentially vulnerable to violent extremism, it is important to shift away from the prevailing risk-oriented approach and to incorporate protective factors more strongly (Lösel et al., 2018), which may protect and/or buffer against radicalization and violent extremism. This may help us explain why people who have similar risk profiles display diverse behavioral outcomes (see Corner et al., 2019 for the concept of multifinality within violent extremism). Notably, this necessitates more research on both direct promotive and buffering protective factors when risk factors are present. Such research is key to better understand vulnerability to violent extremism and when designing successful prevention programs (Borum, 2014). Finally, from a practical perspective, it is key to acknowledge the interactive effects between risk and protective factors and to incorporate direct promotive as well as buffering protective factors more strongly in the design of intervention programs as well as in structured professional judgment risk assessment and management instruments (King et al., 2018).

## Data Availability Statement

The raw data supporting the conclusions of this article will be made available by the authors, without undue reservation.

## Ethics Statement

The studies involving human participants were reviewed and approved by UCL research ethics committee. The patients/participants provided their written informed consent to participate in this study.

## Author Contributions

BR and PG were responsible for the overall conceptualization and designed of this study. BR carried out the statistical analysis. PG secured funding for the study. Both authors were involved in writing the manuscript, revised further drafts of the manuscript, and read and approved the submitted version.

## Conflict of Interest

The authors declare that the research was conducted in the absence of any commercial or financial relationships that could be construed as a potential conflict of interest.

## Publisher’s Note

All claims expressed in this article are solely those of the authors and do not necessarily represent those of their affiliated organizations, or those of the publisher, the editors and the reviewers. Any product that may be evaluated in this article, or claim that may be made by its manufacturer, is not guaranteed or endorsed by the publisher.
